# A machine-learning approach to human ex vivo lung perfusion predicts transplantation outcomes and promotes organ utilization

**DOI:** 10.1038/s41467-023-40468-7

**Published:** 2023-08-09

**Authors:** Andrew T. Sage, Laura L. Donahoe, Alaa A. Shamandy, S. Hossein Mousavi, Bonnie T. Chao, Xuanzi Zhou, Jerome Valero, Sharaniyaa Balachandran, Aadil Ali, Tereza Martinu, George Tomlinson, Lorenzo Del Sorbo, Jonathan C. Yeung, Mingyao Liu, Marcelo Cypel, Bo Wang, Shaf Keshavjee

**Affiliations:** 1 Latner Thoracic Research Laboratories, Toronto General Hospital Research Institute, University Health Network, Toronto, ON Canada; 2 Toronto Lung Transplant Program, Ajmera Transplant Centre, University Health Network, Toronto, ON Canada; 3 Department of Surgery, University of Toronto, Toronto, ON Canada; 4 Institute of Medical Science, University of Toronto, Toronto, ON Canada; 5 Department of Computer Science, University of Toronto, Toronto, ON Canada; 6 Peter Munk Cardiac Centre, University Health Network, Toronto, ON Canada; 7 Department of Medicine, University Health Network, Toronto, ON Canada; 8 Interdepartmental Division of Critical Care Medicine, Medical and Surgical Intensive Care Unit, University Health Network, Toronto, ON Canada; 9 Department of Laboratory Medicine and Pathobiology, University of Toronto, Toronto, ON Canada; 10 Vector Institute, Toronto, ON Canada

**Keywords:** Predictive markers, Computational models, Machine learning, Biomarkers

## Abstract

Ex vivo lung perfusion (EVLP) is a data-intensive platform used for the assessment of isolated lungs outside the body for transplantation; however, the integration of artificial intelligence to rapidly interpret the large constellation of clinical data generated during ex vivo assessment remains an unmet need. We developed a machine-learning model, termed *InsighTx*, to predict post-transplant outcomes using n = 725 EVLP cases. InsighTx model AUROC (area under the receiver operating characteristic curve) was 79 ± 3%, 75 ± 4%, and 85 ± 3% in training and independent test datasets, respectively. Excellent performance was observed in predicting unsuitable lungs for transplantation (AUROC: 90 ± 4%) and transplants with good outcomes (AUROC: 80 ± 4%). In a retrospective and blinded implementation study by EVLP specialists at our institution, InsighTx increased the likelihood of transplanting suitable donor lungs [odds ratio=13; 95% CI:4-45] and decreased the likelihood of transplanting unsuitable donor lungs [odds ratio=0.4; 95%CI:0.16–0.98]. Herein, we provide strong rationale for the adoption of machine-learning algorithms to optimize EVLP assessments and show that InsighTx could potentially lead to a safe increase in transplantation rates.

## Abstract (web summary)

Ex vivo perfusion is a unique platform to study isolated human lungs. Here, authors show that a machine learning model, InsighTx, derived from data generated during ex vivo lung perfusion can accurately predict transplant outcomes and increase organ utilization rates.

## Introduction

Precision medicine for isolated organs has been enabled by the development of ex vivo perfusion systems for the lung^1–5^, liver^6,7^, heart^8,9^, kidney^10–12^, and pancreas^13^. For surgeons, these platforms represent a pragmatic approach to assess the suitability of marginal (non standard) donor organs for transplantation^14,15^. Ex vivo lung perfusion (EVLP) is an established ex vivo assessment technology that aids in the recovery of donor lungs that otherwise would have been discarded^1–5,16^, providing a critical source of viable lungs for patients in need of a transplant. While global lung transplant volumes have increased with EVLP integration, they are still significantly outpaced by the number of people added to the waitlist each year—a problem compounded by the recent pandemic^17^. Although use of EVLP is a possible solution to the organ shortage problem^18^, it is limited by the lack of standardized acceptance criteria regarding when to use an organ for transplant^19,20^. Moreover, EVLP decision-making is largely subjective and involves many measurements performed during ex vivo perfusion which can be daunting for inexperienced EVLP programs^19,20^.

During EVLP, lungs are maintained in a normothermic (37 °C) environment, perfused with an acellular perfusate solution, and ventilated using an ICU-grade lung protective ventilator^1–5^. At present, lung monitoring includes physiological (i.e., gas exchange, compliance, airway pressure), biochemical (i.e., glucose and lactate levels, pH, acid-base chemistry), imaging (i.e., radiographic images, bronchoscopy), and biological measurements (i.e., cytokines and chemokines)^1–5^. In a previous study, we developed the ‘Toronto Lung Score’ based on interleukin-6 (IL-6) and IL-8 protein levels in EVLP perfusate, and used it to profile lung inflammation^21^. Additional studies have demonstrated the association between the severity of specific evaluation parameters during EVLP and patient outcomes;^22–25^ however, these studies failed to holistically evaluate the breadth of potential data derived from EVLP.

While artificial intelligence (AI) and machine learning (ML) have had a significant impact on clinical decision-making in other areas of medicine, they have not yet been thoroughly investigated for use during ex vivo organ perfusion. EVLP is particularly well-suited for ML approaches because the ex vivo data are: (i) restricted to an isolated organ and free of confounding signals from other organ systems; (ii) collected longitudinally for several hours, providing a potential trajectory of improvement or deterioration in organ quality, and (iii) derived from numerous different monitoring systems generating a high volume of data. However, evidence that an AI-guided approach to EVLP decision-making could meaningfully impact organ utilization and post-transplant outcomes has not been demonstrated to date.

**Fig. 1:**
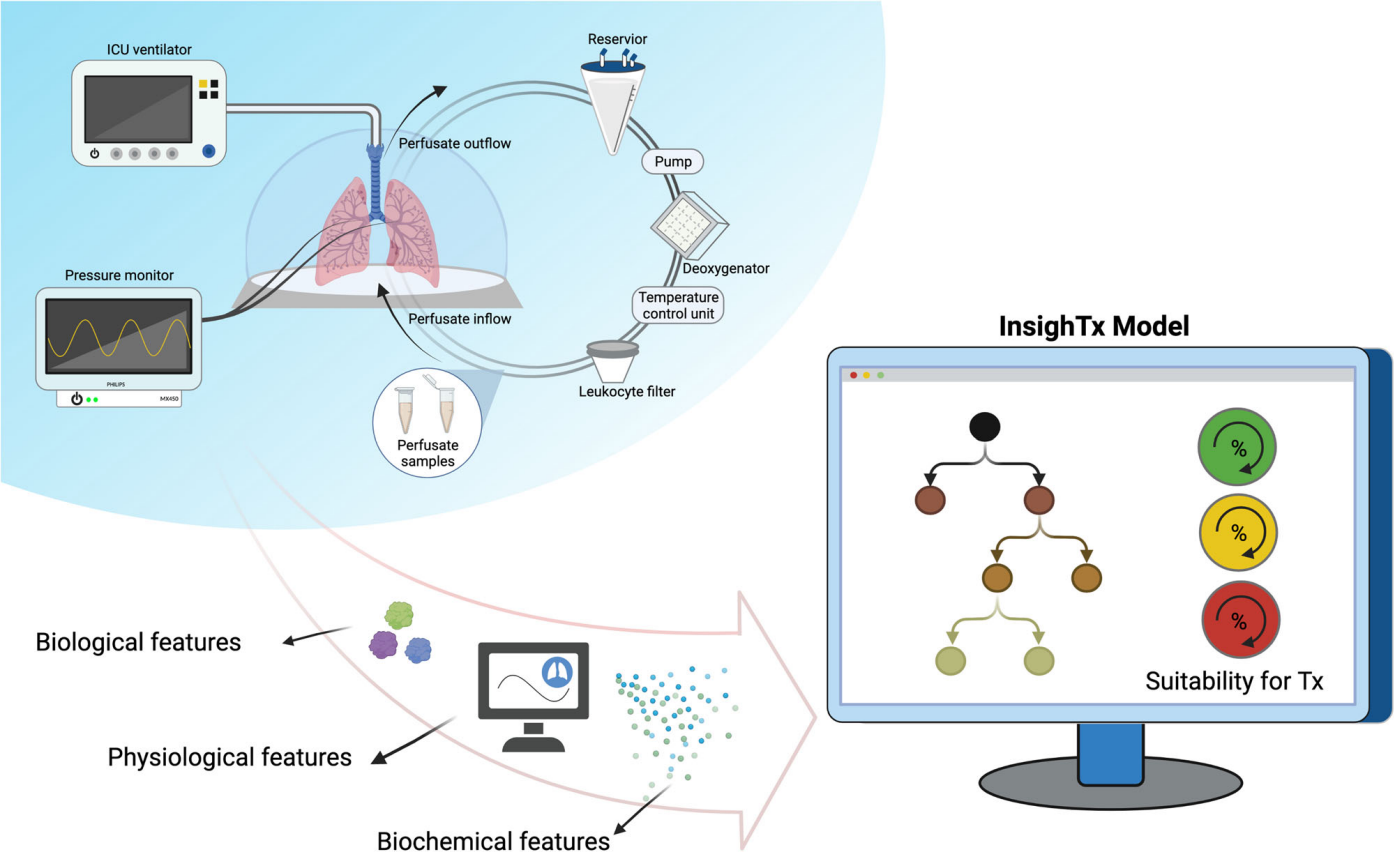
The InsighTx Model. Schematic representation of the development of the InsighTx model using features derived from an ex vivo lung perfusion (EVLP) circuit (top left). Biological, physiological, and biochemical assessments (bottom left) are used as inputs into the XGBoost machine learning algorithm to predict organ suitability for transplant (bottom right). Image created with BioRender.com.

To develop a comprehensive approach to surgical decision-making by leveraging organ assessment data generated during EVLP, we evaluated eXtreme Gradient Boosting (XGBoost)^26^, a decision-tree based ML technique, using clinical EVLP data collected in our center over the past decade. Our ML model, termed InsighTx, uses donor features and all possible assessments made during EVLP to predict suitable lungs for transplantation and patient outcome—the duration of post-transplant mechanical ventilation for the recipient. Important recipient features were then added to donor lung predictions using the InsighTx model to demonstrate an approach that personalizes transplant predictions. We further investigated whether the InsighTx model would impact clinical decision-making during EVLP in a retrospective, real-world evaluation study. This paper summarizes the development of the InsighTx algorithm using the largest collection of clinical EVLP data to date (Fig. 1), and provides evidence that an AI-guided approach could potentially lead to a safe increase in the number of transplants performed following ex vivo assessment.

## Results

### EVLP cohort characteristics

From 2008 to 2022, there were a total of *n* = 725 eligible clinical EVLP cases that were included in InsighTx model development and validation. There were *n* = 504 EVLP cases performed from 2008 to November 2019 that were used as a development dataset. Consecutive EVLP cases conducted between December 2019 to December 2020 (*n* = 97) and December 2020 to August 2022 (*n* = 124) were used as validation cohorts 1 and 2 respectively (Table 1). There were no significant differences in donor age, sex, BMI or type (Table 1); however, the proportion of donation after circulatory death (DCD) compared to donation after brain death (DBD) donors increased in the validation cohorts; median warm ischemic time was 65 min [IQR: 50–80 min]. Transplant rates and post-transplant outcomes significantly varied (Table 1). The rate of transplantation following EVLP was the highest in Test Dataset 1 (66%) and lowest in Test Dataset 2 (49%). While the incidence of Primary Graph Dysfunction (PGD) Grade 3 at 72 h was consistent in this study, we observed that the proportion of patients extubated in less than 72 h was highest in Test Dataset 1 (49%) and lowest in Test Dataset 2 (30%) (Table 1). Although extubation times varied, the median time spent in the ICU was similar across the datasets (Table 1). Of all donor lungs evaluated on EVLP, 38% resulted in transplantation and extubation in less than 72 h post-transplant, 22% were transplanted but associated with prolonged ventilation, and 40% were deemed unsuitable for transplant. These prevalence rates were used as the reference baseline for the area under the precision-recall curve (AUPRC) of EVLP and transplant outcomes.

**Table 1:** Clinical EVLP case characteristics for InsighTx model development

	Training Dataset	Test Dataset 1	Test Dataset 2	*p*-value
Date range	2008–2019	2019–2020	2020–2022	–
Number of cases	504	97	124	–
Mean age (SD) – Years	45 (17)	48 (16)	47 (16)	0.25
Male sex (%)	328 (65%)	62 (64%)	79 (64%)	0.84
Mean BMI (SD)	27.3 (6.5)	27.3 (6.0)	28.7 (7.1)	0.69
Donor type DBD (%)	259 (51%)	48 (49%)	49 (40%)	0.10
EVLP outcome				
Transplanted (%)	313 (62%)	64 (66%)	61 (49%)	0.02
Declined (%)	191 (38%)	33 (34%)	63 (51%)	
Transplant outcome				
Extubated <72 h (%)	190 (38%)	48 (49%)	37 (30%)	0.04
PGD 3 at 72 h (%)	59 (12%)	4 (4%)	12 (10%)	0.08
Median ICU LOS [IQR] - Days	4 [2–10]	4 [2–6]	5 [3–11]	0.17

Statistics: One-way ANOVA test for age and BMI; Kruskal–Wallis test for ICU LOS; Chi-square test for sex, donor type, EVLP outcome, PGD3, and extubation <72 h.

*SD* standard deviation, *BMI* body mass index, *DBD* donation after brain death, *EVLP* ex vivo lung perfusion, *PGD* primary graft dysfunction, *ICU* intensive care unit, *LOS* length of stay, *IQR* interquartile range.

### InsighTx model development and performance

The AUROC for the overall InsighTx model was 79 ± 3%, 75 ± 4%, 85 ± 3% in the training and test sets, respectively (Table 2 and Supplementary Fig. 1). Importantly, discrimination was high for identifying donor lungs on EVLP that resulted in a time to extubation less than 72 h (AUROC: 80 ± 4% (training), 76 ± 6% (test dataset 1), 83 ± 4% (test dataset 2)) and for identifying lungs that were unsuitable for transplantation (AUROC: 90 ± 4% (training), 88 ± 4% (test dataset 1), 95 ± 2% (test dataset 2)). Although the prediction of prolonged time to extubation in transplant recipients was modest in test dataset 1 compared to the training dataset (AUROC: 67 ± 6% (training) vs. 62 ± 9% (test dataset 1)), the model performed well in test dataset 2 (AUROC: 76 ± 6%) (Table 2). Importantly, the precision (positive predictive value) of the model to identify any unsuitable donor lung (i.e., declined for transplant or extubated ≥72 h) was 81% and model precision for suitable donor lungs (i.e., extubated <72 h) was similar at 72%. Furthermore, the AUPRC showed a marked improvement of the InsighTx model to predict EVLP outcomes compared to baseline AUPRC values (prevalence of the respective endpoints) (Supplementary Fig. 1). For patients extubated <72 h (baseline AUPRC 38%), the InsighTx model had an AUPRC of 67 ± 6% in the training dataset, 74 ± 8% in test dataset 1, and 64 ± 10% in test dataset 2. Similar AUPRC results were observed in patients that required prolonged ventilation post-transplant: 40 ± 7% (training), 31 ± 11% (test dataset 1), and 42 ± 11% (test dataset 2) for the InsighTx model vs. 22% for the baseline AUPRC. Notably, the improvement in AUPRC was the strongest for lungs deemed unsuitable for transplant (InsighTx: 86 ± 5% (training), 81 ± 7% (test dataset 1), and 96 ± 2% (test dataset 2) vs. 40% baseline AUPRC).

**Table 2:** AUROC performance of the InsighTx model to predict EVLP and Tx outcomes

	InsighTx Model (Overall)	Extubated <72 h	Extubated ≥72 h	Declined for Tx
**AUROC (SD)**				
Training Dataset	79 (3)	80 (4)	67 (6)	90 (4)
Test Dataset 1	75 (4)	76 (6)	62 (9)	88 (4)
Test Dataset 2	85 (3)	83 (4)	76 (6)	95 (2)
*p*-value^a^	*p* = 0.50	*p* = 0.48	*p* = 0.49	*p* = 0.48
*p*-value^b^	*p* = 0.36	*p* = 0.33	*p* = 0.46	*p* = 0.32

Data reported from the training dataset are derived from the results of the internal test sets. A detailed description of the *p*-value calculations can be found in Methods.

*AUROC* area under receiver operating characteristic curve (%), *SD* standard deviation.

^a^*p*-value for Test Dataset 1 vs. Training Dataset.

^b^*p*-value for Test Dataset 2 vs. Training Dataset.

We further investigated the relationship between the InsighTx model and PGD Grade 3 at 72 h. For donor lungs that were predicted to have a time to extubation <72 h using the InsighTx model, the negative predictive value (NPV) for PGD Grade 3 at 72 h post-transplant was 88% [95% CI: 84–91%, *p* < 0.001, *n* = 430].

A central characteristic of the XGBoost algorithm is the ability to determine the relative importance of the input variables. Only donor type and PEEP (positive end-expiratory pressure) had SHAP (shapley additive explanations) importance values of 0 and were therefore not used by the model for outcome prediction; all other input features were required by the model (SHAP > 0). We observed unique combinations of the donor and EVLP parameters that underlie the prediction of each clinical endpoint (Table 3).

**Table 3:** Ranked EVLP features for endpoint prediction

Rank	Extubated <72 h post-transplant	Extubated ≥72 h post-transplant	Declined for transplant
1	Static Compliance	Static Compliance	∆pO_2_
2	∆pO_2_	Perfusate loss	Static compliance
3	Ca^2+^	Dynamic compliance	Airway pressure
4	Base excess	Airway pressure	Dynamic compliance
5	Dynamic compliance	IL-8	Perfusate loss
6	Perfusate loss	K^+^	Base excess
7	Airway pressure	∆pCO_2_	pH
8	Na^+^	LA pressure	Ca^2+^
9	pH	Na^+^	HCO_3_^−^
10	IL-8	Glucose	∆pCO_2_
11	K+	∆pO_2_	Glucose
12	Donor BMI	IL-6	LA pressure
13	∆pCO_2_	Ca^2+^	Donor age
14	IL-6	Donor sex	Vascular resistance
15	Cl^−^	Base excess	Cl^−^
16	Vascular resistance	pH	K^+^
17	HCO_3_^−^	Vascular resistance	Donor BMI
18	LA pressure	HCO_3_^−^	Lactate
19	Donor sex	PA pressure	IL-10
20	Lactate	Donor age	Na^+^
21	IL-10	Donor BMI	–
22	Glucose	IL-1β	–
23	IL-1β	Cl^−^	–
24	PA pressure	Lactate	–

∆*pO*_*2*_ change in oxygen partial pressure, ∆*pCO*_*2*_ change in carbon dioxide partial pressure, *LA* left atrial, *PA* pulmonary artery, *IL-8* interleukin-8, *IL-6* interleukin-6, *IL-10* interleukin-10, *IL-1β* interleukin-1beta, *BMI* body mass index.

### InsighTx model and recipient features

We investigated whether the inclusion of key recipient features increased the performance of the InsighTx model and the prediction of post-transplant time to extubation. To do this, we employed a sequential modeling approach where the InsighTx results were combined with recipient age, sex, BMI, status, and indication for transplant to generate a secondary, updated probability of post-transplant outcome. As might be expected, the addition of recipient features increased the AUROC for the InsighTx model to discriminate which EVLP cases would result in short or prolonged time to extubation in transplant patients (Supplementary Table 1). A significant increase of 10% in the AUROC was observed compared to a recipient-only model and a similar trend of +6% in AUROC was observed versus the InsighTx model alone (Supplementary Table 1).

### InsighTx implementation analysis

Our analysis showed that the InsighTx model demonstrated good net benefit for transplant suitability and post-transplant extubation <72 h decisions over a wide range of threshold probabilities (Supplementary Fig. 2). As expected, we noted that transplant ‘all’ or ‘none’ approaches were beneficial at the lowest and highest threshold probabilities, respectively (Supplementary Fig. 2), which likely reflects historical transplant decisions based on recipient urgency.

Lastly, we sought to investigate whether the results of the InsighTx model would have a meaningful impact on surgical decision-making during EVLP. A summary of the donor and recipient characteristics for this subset of EVLP cases are provided in Supplementary Table 2.

Overall, we observed that InsighTx model use encouraged a theoretical increase of 7% in the decision to proceed to transplant for lungs more likely to produce good outcomes and a 4% decrease in the decision to proceed to transplant for lungs that were unsuitable (Supplementary Table 3). Interestingly, we observed a net decrease of 13% for the utilization of lungs that resulted in the need for prolonged ventilation, with no change in the lung assessment score (Supplementary Table 3). Most notably, for lungs that were historically declined but predicted to be suitable by InsighTx, there was a 13% increase in decision to proceed to transplant when the ML based decision-aid was available (Supplementary Table 3).

Using a mixed effects logistic regression model, we observed a clinically meaningful impact of InsighTx on surgical decision-making. For lungs that were actually transplanted and had extubation <72 h or which were not transplanted but had a high probability of extubation <72 h on InsighTx, having the InsighTx model available for decision-making resulted in a 13-fold increase [95% CI: 4–45] in the odds of a favorable transplant decision and an improvement of +0.95 [95% CI: 0.4–1.51] in lung suitability assessments (i.e., the impression of lung suitability may have increased from 8 to 9 (out of 10) for a given assessor) (Table 4). Moreover, the opposite was true for unsuitable donor lungs (i.e., decreased odds of transplant and less favorable impression of the organ) (Table 4). When respondents were grouped by EVLP experience level (i.e., number of clinical EVLP cases performed; experience threshold of 100 cases), we observed a consistent effect of the model on decision making (Supplementary Table 4). Notably, those with less EVLP experience tended to have a lower baseline rate of transplantation (Supplementary Table 4).

**Table 4:** Summary of the impact of InsighTx on clinical decision-making

	Transplant decision OR [95% CI]	Clinical impression of donor lung^a^ ∆ [95% CI]
Suitable donor lungs (*n* = 12 lungs)	13 [95% CI: 4–45]	+0.95 [95% CI: 0.4–1.51]
Unsuitable donor lungs (*n* = 8 lungs)	0.4 [95% CI: 0.16–0.98]	−0.31 [95% CI: −0.75 to 0.14]

*OR* odds ratio, *CI* confidence interval.

^a^Assessors were asked to rank the overall impression of a donor lung from poor (0) to excellent (10). Shown are the changes in clinical impression for suitable and unsuitable donor lungs when the InsighTx model was available.

## Discussion

In the present study, we observed that a ML approach to organ assessment predicts EVLP and post-transplant outcomes. The *InsighTx* model was developed using the largest collection of clinical EVLP cases to date and has learned from decisions made by an experienced EVLP program. The model performed extremely well in the prediction of three possible outcomes following EVLP, with an AUROC of 79%, 75%, and 85% in the training and two test datasets, respectively. Furthermore, we demonstrated that the addition of recipient features to InsighTx predictions can be used to further fine-tune model performance. Most importantly, we show that the model represents a surgical decision-aid that could potentially lead to a safe increase in transplant volume at our institution.

An important observation from this study was that InsighTx performance was maintained in all three datasets, spanning over a decade of clinical EVLP practice, even though the prevalence of key clinical outcomes (such as post-transplant extubation <72 h) varied in the cohorts. These results reflect the robust nature of InsighTx to accurately assess the donor lung and predict clinical outcomes, irrespective of different donor populations and time periods. This finding is especially important given that Test Dataset 1 and 2 occurred during the COVID-19 pandemic which impacted lung transplant programs and organ donation rates. Thus, the results herein suggest that the InsighTx model is generalizatable to the evolving landscape of lung transplantation. As with all predictive assays for lung transplantation, future studies that involve periodic validation of clinical accuracy are warranted to continually evalutate the impact of clinical practice evolution.

Studies by our group and others have shown the predictive value of various biomarkers during EVLP^21–25, 27–32^. A study by DiNardo et al. demonstrated that physiological and biochemical features may help to make a decision to transplant^22^. In addition, numerous other studies have highlighted the predictive role of inflammatory cytokines, including IL-6, IL-8, IL-10, and IL-1β, for the assessment of lung injury^21,23–25^. As such, the approach taken in the present study attempts to advance all of the available data and research conducted to date towards the development of a comprehensive and unified ML-based EVLP assessment model. It is important to note that traditional cytokine testing approaches operate on timelines that are not practical for clinical EVLP; however, rapid (i.e., <40 min, TORdx LUNG) cytokine testing platforms (Supplementary Table 5) enable the integration of these features with the InsighTx model. In doing so, previous reports on the importance of biological data can be included in the InsighTx model for real-time decision making. At present, these platforms are restricted to inflammatory cytokines but, as technical capabilities expand, other previously reported protein biomarkers can be added to future iterations of the InsighTx model.

Historically, most studies on EVLP biomarker studies have focused on dichotomous endpoints and, therefore, fail to adequately represent the spectrum of outcomes following EVLP. A unique feature of the InsighTx model is the reporting of the likelihood of three possible clinical outcomes following EVLP. This provides surgeons with a comprehensive view of the most probable recipient outcome post-transplant. Notably, the model showed excellent performance in predicting donor lungs that were: (i) likely to result in a short time to extubation post-transplant, or (ii) unsuitable for transplantation. Moreover, the prediction of post-transplant extubation <72 h by InsighTx was strongly predictive of non-PGD Grade 3 at 72 h. The ability of the InsighTx model to discriminate donor lungs that were associated with prolonged ventilation post-transplant was modest, but showed marked improvement over standard practice. An important finding in our study was that InsighTx precision was 81% when prolonged ventilation and declined for transplantation EVLP cases were considered together. These results strongly support the notion of an injured donor lung phenotype identified by InsighTx which can be used to guide clinical decision-making during EVLP.

The objective of this study was to derive a model for an isolated donor lung to help predict outcome for any recipient, irrespective of their pre-transplant condition or status. It is important to note that the final decision to transplant resides with the surgeon, who takes relevant recipient features into account. Using a donor-centric approach, we observed that a ML model based on donor and EVLP features alone actually demonstrated excellent performance. Although the addition of recipient features to InsighTx improved model AUROC, it did not reach statistical significance, which underscores the importance and good performance of the donor-centric approach of the InsighTx model. Nevertheless, we found that the addition of recipient characteristics, as might be expected, can strengthen model discrimination for post-transplant time-to-extubation.

The sequential donor-recipient modeling approach underscores the power of the InsighTx model: one can use it for donor lung assessment as a generalized model for any recipient or InsighTx results can be combined with specific recipient details that will personalize the prediction to a particular patient. Our results offer further support of the role that the recipient contributes to their post-transplant outcome. For example, recipient age, BMI, and pre-transplant status (urgency) were found to be important modifiers of the InsighTx model predicted outcome. Future studies should investigate additional, more complex recipient features using the approach described herein.

As the field of ex vivo organ perfusion continues to expand, targeted therapies and regenerative strategies will be applied during ex vivo preservation to improve organ function^18^. Thus, the InsighTx model is well-suited to meet this future state by focusing on the outcome of the organ alone, and will be able to better gauge the impact of any future intervention on a donor lung, thereby ensuring that all donor lungs are well conditioned prior to transplant into any recipient.

Detailed analysis of the InsighTx model revealed a different mix of assessment parameters underlying the various endpoint classifications. While this finding was not unexpected, it is interesting to note the relative importance of various features in relation to lung suitability and patient outcomes. Our findings support previous observations that donor type and PEEP (set to a constant for nearly all cases) were unlikely to be associated with outcome and, thus, provide little predictive value. Biological and biochemical biomarkers were highly ranked for the prediction of post-transplant outcome. In particular, we observed that acid-base chemistry was extremely important in determining patient outcomes. Features such as pH and base excess are well known biomarkers of metabolic and respiratory acidosis in lung physiology;^33^ however, the identification and weighting of these markers in EVLP by the InsighTx model is novel and further underscores the value of an AI-based approach to evaluate and understand the significance of ex vivo assessments.

One of the key findings in the present study was the real-world evaluation of the use of the InsighTx model on surgical decision-making. While there have been reports of predictive ML algorithms in thoracic surgery^34^, this is the first such study to show that the use of an AI-based decision-aid during EVLP could theoretically change and improve lung transplant decisions. The results of this study suggest that the impact of ML on transplantation rates could be dramatic and that an overall increase in transplant activity at the program level is plausible. Of note, the effects of the ML model were different based on the predicted post-transplant outcome. For lungs that were associated with poor outcomes, there was a large decrease in the tendency to transplant. This decrease was offset by an even larger increase in the decision to transplant lungs that were historically declined, but predicted by the InsighTx model to have good post-transplant outcomes. These results also demonstrated that experienced EVLP personnel would be more likely to transplant additional donor lungs on EVLP; however, the net gain or decrease in transplantation rates were similar regardless of experience level. Thus, these findings suggest that overall donor lung utilization rates could appropriately and safely increase with InsighTx model implementation for all centers and would likely be of greater benefit to those with less EVLP experience. It is important to note the limitation that this analysis was derived from retrospective adjudication and reflects the views of the participants at our center. Thus, external validation followed by a prospective, multicentre trial is warranted to fully study and understand the broader impact of InsighTx on surgical decision-making and validate our findings that using the InsighTx AI model during EVLP can safely increase transplantation rates.

Although machine learning models can be used to accurately predict medical outcomes, careful consideration regarding the scope and ease in which the data are available will directly impact clinical translation^35^. To that end, the data features used by the InsighTx model are routinely collected and accessible during standard EVLP practice (Summarized in Supplementary Table 5). In the future, the extraction of these data can be automated and directly linked to the InsighTx algorithm, thereby enabling streamlined integration of the model during clinical EVLP in real-time. This approach offers the exciting possibility of leveraging the performance associated with machine learning algorithms while not causing undue burden on clinical EVLP teams.

While the results of this study are promising, there are several limitations to our findings. The model was developed and validated in a cohort of lungs from a single, experienced institution. While this data represents the largest collection of clinical EVLP cases to date, future studies involving large external datasets are needed to confirm our findings. In addition, improvements to expand the breadth of InsighTx biomarkers and data, such as including additional features and/or real-time monitoring and analysis of parameters instead of hourly, is likely to enrich the data quality and strengthen the results of the model. Current efforts are underway to realize this potential.

In conclusion, ex vivo organ perfusion techniques are poised to revolutionize the approach to organ repair, regeneration, and transplantation. While these techniques are being established, a comprehensive and standardized approach to organ assessment is necessary. Using a clinically established ex vivo lung perfusion technique, EVLP, we show that an AI-based ML model is accurate and can safely lead to more transplants. As the number of patients waiting for a transplant continues to grow and outpace the number of available donor organs, the development of novel strategies that maximize the usage of these scarce resources becomes critical. The development of InsighTx to safely identify more viable donor lungs represents a significant step forward for the field of organ perfusion and transplantation by promoting a precision-medicine approach to surgical decision-making.

## Methods

### Study population

Informed consent was obtained from all participants. Institutional approval for this study was obtained (UHN REB#12-5488-13). All consecutive clinical EVLP cases performed at Toronto General Hospital (University Health Network, Toronto, ON, Canada) from 2008 to 2022 were considered for model development and validation. Model training was performed using consecutive clinical EVLP cases occurring between 2008 and November 2019, whereas Test Datasets 1 and 2 represented consecutive cases conducted between December 2019 and December 2020 and December 2020 and August 2022, respectively. Transplant recipient inclusion criteria included adults with end-stage lung disease referred for first lung transplantation. Exclusion criteria were double lung EVLP assessments that resulted in single lung transplantation.

### Data collection and storage

All data were recorded and stored with institutional approval (UHN REB#11-0170-AE). Our EVLP technique has been previously described^1–5^. Briefly, lung assessments are made hourly and data are derived from an ICU-grade ventilator, pressure monitors and perfusate samples collected from the EVLP circuit. Additional features were extracted from the donor chart at the time of EVLP. Biochemical and oxygenation data were generated using a blood gas analyzer (RAPIDPoint, Siemens Healthcare, Germany). ∆pO_2_ and ∆pCO_2_ measurements were calculated as the venous-arterial difference in oxygenation and carbon dioxide partial pressure in perfusate solution, respectively. Protein measurements (i.e., IL-6, IL-8, IL-10, IL-1β) were completed by ELISA (Ella by Protein Simple Inc., San Jose, CA, USA and TORdx LUNG by SQI Diagnostics Inc., Toronto, ON, Canada). A summary of EVLP parameters is provided in Supplementary Table 6 and Supplementary Table 7. Primary Graft Dysfunction (PGD) grades were assigned in accordance with the International Society for Heart and Lung Transplantation working group 2016 definition^36^.

### Data preprocessing

EVLP data were extracted from our Toronto Lung Transplant Program Database and assessed for completeness. Missing data was obtained using the original source documents and records, or accounted for by the XGBoost algorithm during model training and testing. Supplementary Table 5 summarizes parameter source and acquisiton time. For each parameter that was assessed longitudinally during EVLP, the following features were extracted from the data up to four hours: minimum and maximum values, trend during EVLP, and the last recorded value. For EVLP cases that lasted between four and six hours, data was capped after four hours to standardize model predictions. Compliance and protein measurements were normalized to donor lung size using estimated total lung capacity.

### InsighTx model development

A comprehensive list of all assessment features used in model development can be found in Supplementary Table 6. The InsighTx model was developed using a class-weighted XGBoost algorithm (v1.4.2) trained to predict the following clinical endpoints: (i) donor lungs on EVLP deemed unsuitable for transplantation and EVLP cases that resulted in transplantation with recipients who were extubated in (ii) <72 h or (iii) ≥72 h post-transplant. EVLP cases from 2008 to 2019 were used to train the model using donor and EVLP features. The development cohort was used to establish the model hyperparameters and randomly partitioned 80:20 for training and testing―five-fold cross-validation was performed where one-fold was used as the internal test set at each of the five iterations (Note: data reported from the development cohort are derived from the results of the internal test sets). Data arising from EVLP cases conducted from 2019 to 2020 and 2020 to 2022 were used as two independent validation cohorts to test the InsighTx model. Each EVLP case was assigned a predicted outcome based on the endpoint with the highest probability (most likely outcome) derived from the InsighTx model. Predicted outcomes were used for model performance analyses and in the implementation study analysis.

### InsighTx and recipient model development

A random forest model was used to evaluate the addition of recipient features (age, sex, body mass index (BMI), patient status^37^, and indication for transplant) to the outcome probabilities of the InsighTx model. Recipient status was recorded at assessment, listing, and transplant admission according to standard procedures at our institution^37^. All EVLP cases that resulted in bilateral transplantation (*n* = 368) were included, and five-fold cross validation was performed. Supplementary Table 8 lists the summary statistics for recipient features used in this analysis.

### Implementation analysis

**Fig. 2:**
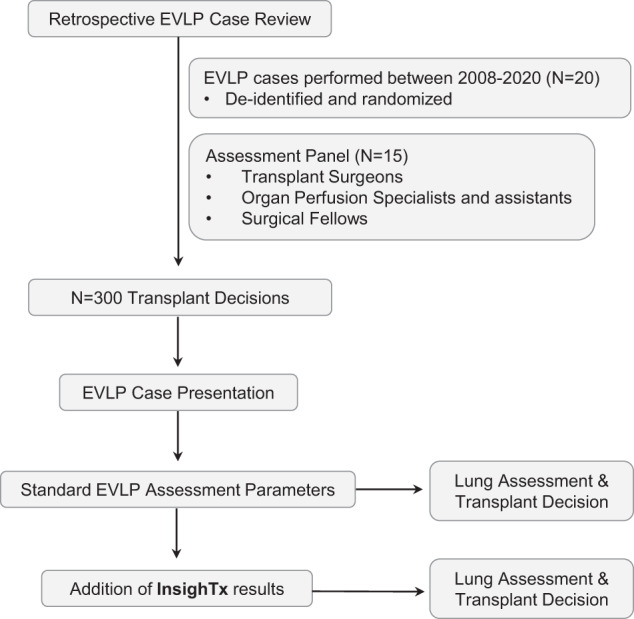
Clinical Review Schematic. Schematic for the retrospective EVLP case review with InsighTx.

To evaluate the effect of InsighTx on clinical decision-making, we conducted a blinded retrospective case review for a subset of *n* = 20 EVLP cases in this study, with a panel of *n* = 15 participants comprising surgeons (*n* = 7), surgical fellows (*n* = 3), organ perfusion specialists (*n* = 3), and EVLP assistants (*n* = 2) at our institution (Fig. 2). Each case was de-identified and presented alongside donor and recipient information. For declined EVLP cases, the details of the intended recipient were used. The study cases included: six cases where the historical outcome matched the InsighTx model prediction (i.e., extubated <72 h or declined for transplant), nine cases that were historically declined for transplant but the InsighTx model predicted that the lungs were likely to produce a good transplant outcome, and five lungs where the InsighTx model correctly predicted the need for prolonged ventilation. For statistical analysis, cases were grouped as either suitable (predicted time to extubation <72 h) or unsuitable (predicted time to extubation ≥72 h or declined for transplant) for transplantation. Assessors were asked to determine the suitability of the lung for transplant (yes or no) based on standard EVLP evaluation parameters alone and their assessment (impression) of the organ on a scale from 0 (poor) to 10 (excellent). The predicted transplant outcome from the InsighTx model was then revealed and respondents were asked to re-answer the transplant suitability and lung assessment questions. This study analysis was reviewed and approved by our institution (UHN REB#19-6251).

### Statistical methods

Demographic and clinical data were summarized using descriptive statistics for the development and testing cohorts and compared using Chi-squared, ANOVA, and Kruskal–Wallis tests. The area under the receiver operating characteristic (AUROC) and precision-recall (AUPRC) curves were used to assess the predictive performance of the overall InsighTx model as well as each clinical outcome of interest. Training and Test Dataset *p*-values were determined using bootstrapping. Briefly, using the Test Dataset sample size, a subset of datapoints were randomly selected from the Training Dataset and the AUROC of the subset was obtained from the model predictions. This was repeated 10,000 times to generate an underlying distribution of the AUROC values. The respective AUROC value from the Test Dataset was then used to determine a cutoff for the distribution, and the proportion of data in the distribution less or greater than the Test Dataset AUROC was used to determine the *p*-value. Net benefit analysis was conducted using the decision to transplant or time-to-extubation as a binary outcome on all study cases. InsighTx model net benefit was compared to transplant ‘all’ or ‘none’ approaches. To further estimate the effect of the InsighTx model results on decision-making in the retrospective review, a logistic regression model was fitted, with the suitability for transplant as the outcome, fixed effects for use of InsighTx, and EVLP group, and random effects for study case and assessor. All analyses were conducted using Stata (StataCorp, College Station, TX, USA), GraphPad (GraphPad Software, San Diego, CA, USA), SPSS Statistics (IBM Corp, Armonk, NY, USA), Python Programming Language (Python Software (v3.9), Wilmington, DE, USA), or R statistics software (R Foundation for Statistical Computing, Vienna, Austria).

### Reporting summary

Further information on research design is available in the Nature Portfolio Reporting Summary linked to this article.

### Supplementary information

Supplementary Information File

Reporting Summary

### Source data

Source Data

## Data Availability

All data supporting the findings described in this manuscript are available in the article, Supplementary Information File, and from the corresponding authors upon request. A Source Data file has also been provided. Our study design did not include provisions to share the de-identified individual participant data, given historical concerns from our institution’s Research Ethics Board on the inherent risk of potentially identifying a participant using a combination of de-identified data fields. Thus, individual patient data from this study will not be made available in publicly accessible databases. However, researchers affiliated with accredited research institutions may request access by contacting the corresponding authors (S.K. and B.W.) who will respond within one month of the request. Data transfer and usage restrictions will be in accordance with the data sharing agreement policies and procedures at University Health Network. Source data are provided with this paper.

## Code availability

The study design approved by our institution did not include provisions to share source InsighTx code from this study and it is not available in publicly accessible databases. However, researchers affiliated with accredited research institutions may request access by contacting the corresponding authors (S.K. and B.W.) who will respond within one month of the request. Code transfer and usage restrictions will be in accordance with the data and material sharing agreement policies and procedures at University Health Network. A detailed description of the InsighTx model using XGBoost can be found via GitHub (https://github.com/bowang-lab).
