# Supplementary material for: A machine-learning approach to human ex vivo lung perfusion predicts transplantation outcomes and promotes organ utilization
Source: Nat Commun. 2023 Aug 9;14:4810. doi: 10.1038/s41467-023-40468-7 (PMC10412608; doi:10.1038/s41467-023-40468-7)
Supplement: Supplementary file 1 — Supplementary Information File [file 41467_2023_40468_MOESM1_ESM.pdf]

Supplementary Information File:

A machine-learning approach to human ex vivo lung perfusion predicts transplantation outcomes and promotes organ utilization

Table of Contents:

Supplementary Figures...Page 2
Figure 1: InsighTx model performance on test data
Figure 2: Net Benefit of the InsighTx model

Supplementary Tables...Page 4
Table 1: Performance (AUROC) of donor and/or recipient models that predict time to extubation in transplanted patients
Table 2: Recipient and donor characteristics for InsighTx model assessment
Table 3: Summary of the theoretical impact of InsighTx on organ utilization and impression
Table 4: Summary of the theoretical transplantation rates by EVLP experience with and without InsighTx
Table 5: Summary of source data acquisition for the InsighTx model
Table 6: All EVLP features used in the InsighTx model
Table 7: Summary of quantitative data derived from EVLP assessment features
Table 8: Transplant patient characteristics for InsighTx with recipient features model

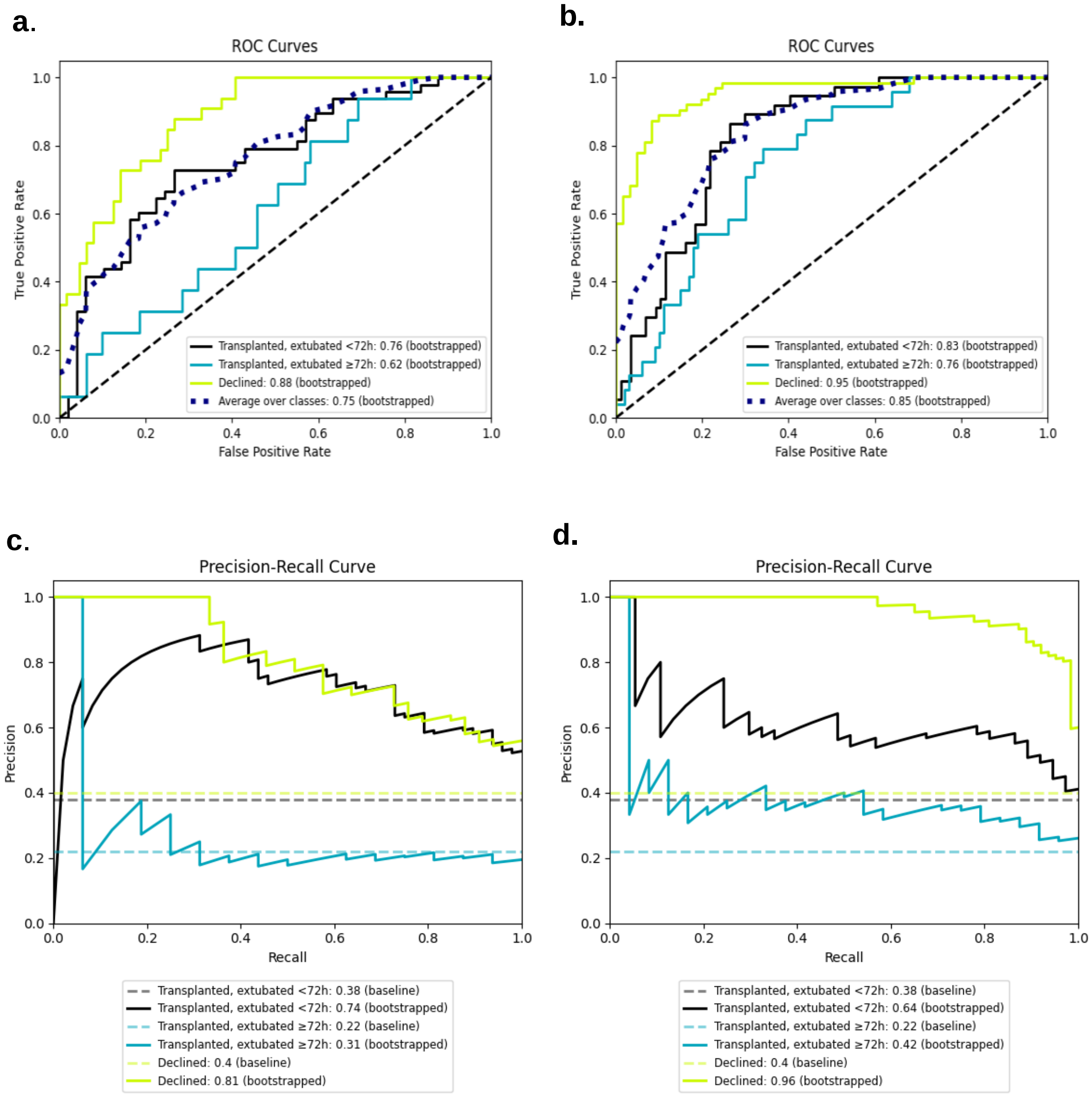

Supplementary Figure 1: **InsighTx model performance on test data**. AUROC graphs for the InsighTx model performance in Test Dataset 1 (a) and Test Dataset 2 (b). Shown are the AUROCs for the overall model (dotted blue line), prediction of post-transplant extubation <72h (black line), ≥72h (blue line), and unsuitable for transplant (yellow line). The dashed line represents an AUROC of 50%. Panel (c) and (d) show the AUPRC graphs for the InsighTx model performance in Test Dataset 1 and Test Dataset 2, respectively. Shown are the AUPRCs for the prediction of post-transplant extubation <72h (black line), ≥72h (blue line), and unsuitable for transplant (yellow line). The dashed lines represent the baseline AUPRC for each outcome. Bootstrapped AUROC and AUPRC values are reported in the respective figure legends. Source data are provided as a Source Data file.

a. b.

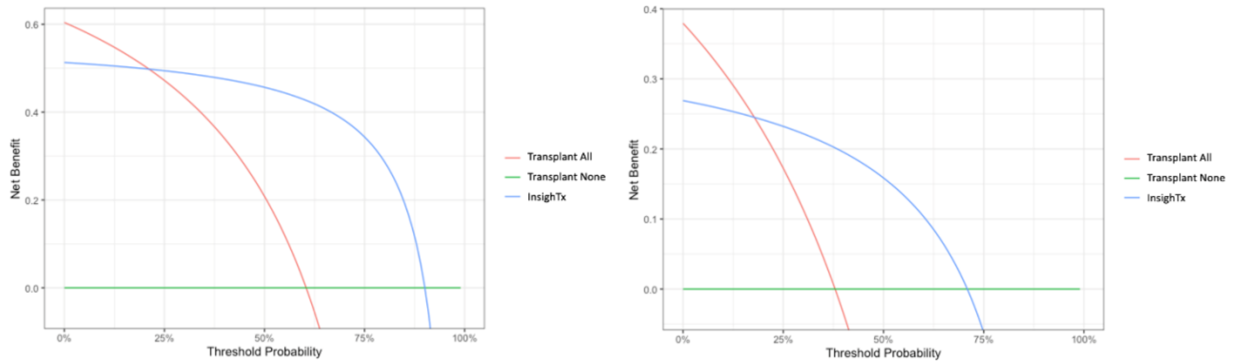

Supplementary Figure 2: **Net Benefit of the InsighTx model**. Net benefit graphs of the InsighTx model (blue trace) compared to transplant ‘all’ (red trace) or ‘none’ (green trace) approaches to predict transplant suitability (a) or post-transplant extubation <72h (b). Source data are provided as a Source Data file.

Supplementary Table 1: Performance (AUROC) of donor and/or recipient models that predict time to extubation in transplanted patients

Model	AUROC (SD)
InsighTx + Recipient Features	79 (4)
InsighTx	73 (8)
Recipient Features Only	69 (6)
p-value[1]	0.17
p-value[2]	0.01

Legend: 1: p-value for “InsighTx + Recipient Features” vs. “InsighTx”; 2: p-value for “InsighTx + Recipient Features” vs. “Recipient Features”; AUROC=area under receiver operating characteristic curve (%); SD=standard deviation. A detailed description of the p-value calculations can be found in the Methods.

Supplementary Table 2: Recipient and donor characteristics for InsighTx model assessment

	**Study Cases**
Date Range	2008-2020
Number of Cases	20
Mean Age (SD) – Years	
Donor	45 (16)
Recipient	57 (12)
Male Sex (%)	
Donor	13 (65%)
Recipient	12 (60%)
Donor Type DBD (%)	10 (50%)
Recipient Status (%)	
1	6 (30%)
2	10 (50%)
3	4 (20%)
Recipient Disease (%)	
Emphysema/COPD	6 (30%)
Cystic Fibrosis	2 (10%)
PF/ILD/UIP/NSIP	10 (50%)
Other	2 (10%)
EVLP Outcome	
Transplanted (%)	8 (40%)
Declined (%)	12 (60%)
Transplant Outcome	
Extubated <72h (%)	3 (38%)
PGD 3 at 72h (%)	2 (25%)
ICU LOS [IQR] - Days	5 [3-7]

Legend: SD=standard deviation; DBD=donation after brain death; EVLP=ex vivo lung perfusion; PGD=primary graft dysfunction; ICU=intensive care unit; LOS=length of stay; IQR=interquartile range; PF=pulmonary fibrosis; ILD=interstitial lung disease; UIP=usual interstitial pneumonia; NSIP=nonspecific interstitial pneumonia; COPD=chronic obstructive pulmonary disease.

Supplementary Table 3: Summary of the theoretical impact of InsighTx on organ utilization and impression

			**InsighTx vs. SOC**	
**Historical Outcome**	**Predicted Outcome**	**No. Decisions**	**∆Transplant Decisions**	**∆Clinical Impression of Donor Lungǂ**
Extubated <72h Post-Transplant	*Extubated <72h Post-Transplant*	45	+7% (+3 decisions to Tx)	+1.0
Unsuitable for Transplant	*Unsuitable for Transplant*	45	-4% (-2 decisions to Tx)	-1.0
Extubated ≥72h Post-Transplant	*Extubated ≥72h Post-Transplant*	75	-13% (-10 decisions to Tx)	0
Unsuitable for Transplant	*Extubated <72h Post-Transplant*	135	+13% (+18 decisions to Tx)	+0.5

ǂ Assessors were asked to rank the overall impression of a donor lung from poor (0) to excellent (10). Shown are the changes in clinical impression for suitable and unsuitable donor lungs when the InsighTx model was available. Legend: SOC=standard of care; EVLP=ex vivo lung perfusion.

Supplementary Table 4: Summary of the theoretical transplantation rates (%) by EVLP experienceǂ with (+) and without (-) InsighTx

**EVLP Experience**	**Unsuitable Donor Lungs**		**Suitable Donor Lungs**	
	- InsighTx	*+ InsighTx*	*- InsighTx*	*+ InsighTx*
100+	74%	66%	54%	70%
<100	55%	38%	43%	54%

ǂ : experience defined as the number of clinical EVLP cases performed by the assessor – a threshold of 100 cases was used.

Supplementary Table 5: Summary of source data acquisition for the InsighTx model

Input Feature	Data Source	Data Recording	Acquisition Time	Data Notes
Donor characteristics	Patient charts	Text	Instantaneous	N/A
Physiological (i.e., compliance, airway pressure)	ICU grade ventilator (i.e., Maquet Servo-i or similar)	Continuous monitoring	Instantaneous	Recorded hourly
Physiological (i.e., PA and LA pressure)	Pressure monitor (i.e., Philips IntelliVue MX450)	Continuous monitoring	Instantaneous	Recorded hourly
Physiological (i.e., gas exchange)	Perfusate sampling and ABG analysis (i.e., Siemens Rapid-point or similar)	As samples are drawn	<5 minutes	Samples drawn during hourly assessment or as needed
Biochemical (i.e., acid base chemistry, electrolytes)	Perfusate sampling and ABG analysis (i.e., Siemens Rapid-point or similar)	As samples are drawn	<5 minutes	Samples drawn during hourly assessment or as needed
Biological (i.e., cytokines)	Perfusate sampling and ELISA-based analysis (i.e., SQI TORdx LUNG or similar)	Batched sampling during EVLP	<40 minutes	Samples drawn during EVLP to capture cytokine profile
Edema (i.e., perfusate loss and volume exchanges)	Perfusate reservoir	Manual recording	Instantaneous	Annotated hourly by Organ Perfusion Specialists

Legend: LA=left atrial; PA=pulmonary artery; ABG=arterial blood gas

Supplementary Table 6: All EVLP features used in the InsighTx model

Donor	Physiological	Biochemical	Biological
Age	ΔpO_2	Ca^{2+}	IL-10
Sex	ΔpCO_2	Cl^-	IL-1β
BMI	Dynamic Compliance	K^+	IL-6
Donor Type (DBD vs DCD)	Static Compliance	Na^+	IL-8
	PA & LA Pressure	Base Excess	
	Vascular Resistance	HCO_3^-	
	Airway Pressure (Peak, Mean, Plateau)	pH	
	PEEP	Glucose	
	Volume of perfusate loss and exchange	Lactate	

Legend: ΔpO_2=change in oxygen partial pressure; ΔpCO_2=change in carbon dioxide partial pressure; LA=left atrial; PA=pulmonary artery; IL-8=interleukin-8; IL-6=interleukin-6; IL-10=interleukin-10; IL-1β=interleukin-1beta; BMI=body mass index; DBD=donation after brain death; DCD=donation after cardiac death; PEEP=positive end-expiratory pressure.

Supplementary Table 7: Summary of quantitative data derived from EVLP assessment featuresƗ

Parameter	N Data Points	Median [IQR]
Physiological		
ΔpO_2 (mmHg)	2,720	437 [377 – 485]
ΔpCO_2 (mmHg)	2,442	-6 [(-7) – (-4)]
Dynamic Compliance (mL/cmH_2O)	2,654	73 [53 – 89]
Static Compliance (mL/cmH_2O)	2,604	114 [82 – 147]
LA Pressure (mmHg)	6,010	4 [4 – 5]
PA Pressure (mmHg)	6,011	7 [6 – 8]
Pulmonary Vascular Resistance (dynes/sec/cm^5)	5,654	175 [103 – 296]
Peak Pressure (cmH_2O)	5,354	13 [12 – 15]
Plateau Pressure (cmH_2O)	2,655	11 [10 – 13]
Mean Pressure (cmH_2O)	5,327	7 [7 – 8]
PEEP (cmH_2O)	6,733	5 [5 – 5]
Perfusate addition (mL)	2,601	250 [250 – 500]
Perfusate removal (mL)	1,795	250 [125 – 250]
Edema (perfusate loss) (mL)	1,481	90 [50 – 175]
Biochemical		
Ca^{2+} (mmol/L)	2,474	0.87 [0.84 – 0.91]
Cl^- (mmol/L)	2,610	125 [123 – 127]
K^+ (mmol/L)	2,685	4.3 [4.1 – 4.6]
Na^+ (mmol/L)	2,585	150 [148 – 153]
Base Excess (mmol/L)	2,638	-24 [(-27) – (-21)]
HCO_3^- (mmol/L)	2,632	5.9 [4.3 – 7.6]
pH	2,444	7.08 [6.95 – 7.19]
Glucose (mmol/L)	3,639	7.5 [6.0 – 8.6]
Lactate (mmol/L)	2,186	5.8 [3.8 – 8.3]
Biological		
IL-6 (pg/mL)	1,261	4,758 [707 – 18,850]
IL-8 (pg/mL)	1,259	467 [119 – 2,932]
IL-1β (pg/mL)	766	5.4 [1.7 – 15.0]
IL-10 (pg/mL)	756	76.1 [12.8 – 172.8]

Legend: Ɨ: median proportion of cases with a missing parameter was 15%; ΔpO_2=change in oxygen partial pressure; ΔpCO_2=change in carbon dioxide partial pressure; LA=left atrial; PA=pulmonary artery; IL-8=interleukin-8; IL-6=interleukin-6; IL-10=interleukin-10; IL-1β=interleukin-1beta; PEEP=positive end-expiratory pressure.

Supplementary Table 8: Transplant patient characteristics for InsighTx with recipient features model

Number of Cases	368
Mean Age (SD) – Years	55 (14)
Sex (%)	
Female	135 (37%)
Male	233 (63%)
Mean BMI (SD)	24 (5)
Status at Assessment (%)	
1	197 (53%)
2	131 (36%)
3	40 (11%)
Status at Listing (%)	
1	197 (53%)
2	130 (36%)
3	41 (11%)
Status at Admission (%)	
1	116 (32%)
2	139 (37%)
3	113 (31%)
Indication for Transplant (%)	
PF/ILD/UIP/NSIP	161 (44%)
Emphysema/COPD	131 (36%)
Cystic Fibrosis	43 (12%)
Primary pulmonary hypertension	15 (4%)
Retransplant	16 (4%)
Other	2 (<1%)

Legend: SD=standard deviation; BMI=body mass index; PF=pulmonary fibrosis; ILD=interstitial lung disease; UIP=usual interstitial pneumonia; NSIP=nonspecific interstitial pneumonia; COPD=chronic obstructive pulmonary disease.
