# Supplementary material for: A machine-learning approach to human ex vivo lung perfusion predicts transplantation outcomes and promotes organ utilization
Source: Nat Commun. 2023 Aug 9;14:4810. doi: 10.1038/s41467-023-40468-7 (PMC10412608; doi:10.1038/s41467-023-40468-7)
Supplement: Supplementary file 2 — Reporting Summary [file 41467_2023_40468_MOESM2_ESM.pdf]

nature portfolio

Corresponding author(s): Dr. Shaf Keshavjee

Last updated by author(s): Jun 5, 2023

Reporting Summary

Nature Portfolio wishes to improve the reproducibility of the work that we publish. This form provides structure for consistency and transparency in reporting. For further information on Nature Portfolio policies, see our Editorial Policies and the Editorial Policy Checklist.

Statistics

For all statistical analyses, confirm that the following items are present in the figure legend, table legend, main text, or Methods section.

n/a	Confirmed	
[ ]	[x]	The exact sample size (*n*) for each experimental group/condition, given as a discrete number and unit of measurement
[ ]	[x]	A statement on whether measurements were taken from distinct samples or whether the same sample was measured repeatedly
[ ]	[x]	The statistical test(s) used AND whether they are one- or two-sided<br>*Only common tests should be described solely by name; describe more complex techniques in the Methods section.*
[ ]	[x]	A description of all covariates tested
[ ]	[x]	A description of any assumptions or corrections, such as tests of normality and adjustment for multiple comparisons
[ ]	[x]	A full description of the statistical parameters including central tendency (e.g. means) or other basic estimates (e.g. regression coefficient) AND variation (e.g. standard deviation) or associated estimates of uncertainty (e.g. confidence intervals)
[ ]	[x]	For null hypothesis testing, the test statistic (e.g. *F*, *t*, *r*) with confidence intervals, effect sizes, degrees of freedom and *P* value noted<br>*Give P values as exact values whenever suitable.*
[x]	[ ]	For Bayesian analysis, information on the choice of priors and Markov chain Monte Carlo settings
[x]	[ ]	For hierarchical and complex designs, identification of the appropriate level for tests and full reporting of outcomes
[x]	[ ]	Estimates of effect sizes (e.g. Cohen's *d*, Pearson's *r*), indicating how they were calculated

Our web collection on statistics for biologists contains articles on many of the points above.

Software and code

Policy information about availability of computer code

Data collection	Data fields were collected by the Transplant Team at the Toronto General Hospital and stored in an in-house database (Toronto Lung Transplant Program Database, not publicly available). This is described in the methods; please refer to "Data collection and storage" and "Data preprocessing" for additional information.
Data analysis	The data in this study was analyzed using open source code, based on the XGBoost framework (v1.4.2). Reference to this code is provided in the manuscript (Reference 26). A Code Sharing statement has been provided: The study design approved by our institution did not include provisions to share source InsighTx code from this study and it is not available in publicly accessible databases. However, researchers affiliated with accredited research institutions may request access by contacting the corresponding authors (Dr. S. Keshavjee and Dr. B. Wang) who will respond within one month of the request. Code transfer and usage restrictions will be in accordance with the data and material sharing agreement policies and procedures at University Health Network. A detailed description of the InsighTx model using XGBoost can be found via GitHub (https://github.com/bowang-lab).

For manuscripts utilizing custom algorithms or software that are central to the research but not yet described in published literature, software must be made available to editors and reviewers. We strongly encourage code deposition in a community repository (e.g. GitHub). See the Nature Portfolio guidelines for submitting code & software for further information.

Data

Policy information about availability of data

All manuscripts must include a data availability statement. This statement should provide the following information, where applicable:

- Accession codes, unique identifiers, or web links for publicly available datasets
- A description of any restrictions on data availability
- For clinical datasets or third party data, please ensure that the statement adheres to our policy

All data supporting the findings described in this manuscript are available in the article, Supplementary Information File, and from the corresponding authors upon request. A Source Data file has also been provided. Our study design did not include provisions to share the de-identified individual participant data, given historical concerns from our institution's Research Ethics Board on the inherent risk of potentially identifying a participant using a combination of de-identified data fields. Thus, individual patient data from this study will not be made available in publicly accessible databases. However, researchers affiliated with accredited research institutions may request access by contacting the corresponding authors (Dr. S. Keshavjee and Dr. B. Wang) who will respond within one month of the request. Data transfer and usage restrictions will be in accordance with the data sharing agreement policies and procedures at University Health Network.

Human research participants

Policy information about studies involving human research participants and Sex and Gender in Research.

Reporting on sex and gender	Appropriate use of sex is reported throughout the manuscript. Gender information relied on the voluntary supply of this data as part of the characterized clinical data. Given that our study dates back to 2008 we do not have reliable access to this data field and subgroup reporting based on gender is not feasible and there for not reported.
Population characteristics	From 2008 to 2022, there were a total of n=725 eligible clinical EVLP cases that were included in InsighTx model development and validation. There were n=504 EVLP cases performed from 2008 to November 2019 that were used as a development dataset. Consecutive EVLP cases conducted between December 2019 to December 2020 (n=97) and December 2020 to August 2022 (n=124) were used as validation cohorts 1 and 2 respectively (Table 1). There were no significant differences in donor age, sex, BMI or type (Table 1); however, the proportion of donation after circulatory death (DCD) compared to donation after brain death (DBD) donors increased in the validation cohorts; median warm ischemic time was 65 minutes [IQR: 50-80 minutes]. Transplant rates and post-transplant outcomes significantly varied (Table 1). The rate of transplantation following EVLP was the highest in Test Dataset 1 (66%) and lowest in Test Dataset 2 (49%). While the incidence of Primary Graph Dysfunction (PGD) Grade 3 at 72h was consistent in this study, we observed that the proportion of patients extubated in less than 72h was highest in Test Dataset 1 (49%) and lowest in Test Dataset 2 (30%) (Table 1). Although extubation times varied, the median time spent in the ICU was similar across the datasets (Table 1). Of all donor lungs evaluated on EVLP, 38% resulted in transplantation and extubation in less than 72h post-transplant, 22% were transplanted but associated with prolonged ventilation, and 40% were deemed unsuitable for transplant. These prevalence rates were used as the reference baseline for the area under the precision-recall curve (AUPRC) of EVLP and transplant outcomes.
Recruitment	All consecutive clinical EVLP cases performed at Toronto General Hospital (University Health Network, Toronto, ON, Canada) from 2008-2022 were considered for model development and validation.
Ethics oversight	Informed consent was obtained from all participants. Institutional approval for this study was obtained (UHN REB#12-5488-13). All data were recorded and stored with institutional approval (UHN REB#11-0170-AE).

Note that full information on the approval of the study protocol must also be provided in the manuscript.

Field-specific reporting

Please select the one below that is the best fit for your research. If you are not sure, read the appropriate sections before making your selection.

☒ Life sciences ☐ Behavioural & social sciences ☐ Ecological, evolutionary & environmental sciences

For a reference copy of the document with all sections, see nature.com/documents/nr-reporting-summary-flat.pdf

Life sciences study design

All studies must disclose on these points even when the disclosure is negative.

Sample size	All consecutive clinical EVLP cases performed at Toronto General Hospital (University Health Network, Toronto, ON, Canada) from 2008-2022 were considered for model development and validation.
Data exclusions	Transplant recipient inclusion criteria included adults with end-stage lung disease referred for first lung transplantation. Exclusion criteria were double lung EVLP assessments that resulted in single lung transplantation.
Replication	Cross-validation and test dataset validation were performed and described throughout the study.

Randomization	Model training was performed using consecutive clinical EVLP cases occurring between 2008-November 2019, whereas Test Datasets 1 and 2 represented consecutive cases conducted between December 2019 – December 2020 and December 2020 – August 2022, respectively.
Blinding	To evaluate the effect of InsighTx on clinical decision-making, we conducted a blinded retrospective case review for a subset of n=20 EVLP cases in this study, with a panel of n=15 participants comprising surgeons (n=7), surgical fellows (n=3), organ perfusion specialists (n=3), and EVLP assistants (n=2) at our institution (Fig. 2). Each case was de-identified and presented alongside donor and recipient information.

Reporting for specific materials, systems and methods

We require information from authors about some types of materials, experimental systems and methods used in many studies. Here, indicate whether each material, system or method listed is relevant to your study. If you are not sure if a list item applies to your research, read the appropriate section before selecting a response.

Materials & experimental systems

n/a	Involved in the study	
☒	☐	Antibodies
☒	☐	Eukaryotic cell lines
☒	☐	Palaeontology and archaeology
☒	☐	Animals and other organisms
☒	☐	Clinical data
☒	☐	Dual use research of concern

Methods

n/a	Involved in the study	
☒	☐	ChIP-seq
☒	☐	Flow cytometry
☒	☐	MRI-based neuroimaging
